# Mimicry of Central-Peripheral Immunity in Alzheimer's Disease and Discovery of Neurodegenerative Roles in Neutrophil

**DOI:** 10.3389/fimmu.2019.02231

**Published:** 2019-09-25

**Authors:** Joseph Park, Sung Hoon Baik, Inhee Mook-Jung, Daniel Irimia, Hansang Cho

**Affiliations:** ^1^The Nanoscale Science Program, Department of Mechanical Engineering and Engineering Science, Department of Biological Sciences, Center for Biomedical Engineering and Science, University of North Carolina at Charlotte, Charlotte, NC, United States; ^2^Department of Biochemistry and Biomedical Sciences, Seoul National University, Seoul, South Korea; ^3^Department of Surgery, BioMEMS Resource Center, Harvard Medical School, Massachusetts General Hospital, Charlestown, MA, United States; ^4^Department of Biophysics, Institute of Quantum Biology, Sungkyunkwan University, Suwon, South Korea

**Keywords:** neuroinflammation, neurodegeneration, Alzheimer's disease, microglia, neutrophil, chemotaxis, cellular interaction, microfluidics

## Abstract

Neuroinflammatory roles of central innate immunity in brain parenchyma are well-regarded in the progression of neurodegenerative disorders including Alzheimer's disease (AD), however, the roles of peripheral immunity in central nervous system (CNS) diseases are less clear. Here, we created a microfluidic environment of human AD brains: microglial neuroinflammation induced by soluble amyloid-beta (Abeta), a signature molecule in AD and employed the environment to investigate the roles of neutrophils through the central-peripheral innate immunity crosstalk. We observed that soluble Abeta-activated human microglial cells produced chemoattractants for neutrophils including IL6, IL8, CCL2, CCL3/4, CCL5 and consequently induced reliable recruitment of human neutrophils. Particularly, we validated the discernable chemo-attractive roles of IL6, IL8, and CCL2 for neutrophils by interrupting the recruitment with neutralizing antibodies. Upon recruitment, microglia-neutrophils interaction results in the production of inflammatory mediators such as MIF and IL2, which are known to up-regulate neuroinflammation in AD. We envision that targeting the crosstalk between central-peripheral immune community is a potential strategy to reduce immunological burdens in other neuroinflammatory CNS diseases.

## Introduction

Alzheimer's disease (AD) is the most common form of dementia. It affects more than 35 million people worldwide (1, 2). It is characterized by neuropathological features including amyloid-beta (Abeta) plaques, neurofibrillary tangles, and neuroinflammation that lead into synaptic dysfunction and a progressive deterioration of cognitive functions (2–5). A growing body of evidence suggests that neuroinflammation is the critical hallmark of AD and central innate immune cells (microglia and astrocytes) have the major roles of neuroinflammation in a central nervous system (CNS) (6–9). Particularly, microglia, a macrophage resident in brains, produce both beneficial neuroinflammatory responses and detrimental neurotoxic effects in the progression of AD (10–12). Therefore, the regulation of microglial activation has been proposed as a potential therapeutic approach in AD treatment (13).

In contrast to the field's increasing understanding of the neuroinflammatory roles of central innate immunity in AD, comparatively little is known about their interactions with peripheral immunity. Peripheral immune cells including T-cells, B-cells, monocytes, and neutrophils, have been found in the brains of AD human patients and corresponding AD animal models (14–17). Among them, neutrophils are the body's most abundant and frontline immune cells, respond with both central and peripheral immune effectors during the progression of AD (17). The migration of neutrophils is initiated by chemokine-triggered activation. Neutrophils actively crawl through the endothelium and accumulate in tissues where they released neutrophil extracellular traps (NETs) and IL-17 which are markers of sterile inflammation and toxic cytokines, respectively. Neutrophil depletion or inhibition of trafficking *via* LFA-1 blockade reduced Alzheimer's disease–like neuropathology and improved memory in mice showing cognitive dysfunction in transgenic Alzheimer's disease mouse models (18). However, the mechanism study of neutrophil recruitment and induction of neuropathological changes of AD still remains unclear. The challenge of the study is due to, in part, the limited readouts *in vivo*, the similar profiles of peripheral inflammatory roles compared to the central immunity by microglia and astrocytes, and inconsistent observation and/or variation in AD animal models. Recently, microfluidic organ-on-chips demonstrated successfully to provide feasible, regulated, accessible microenvironments to study multicellular interaction and/or multiorgan behaviors in quantitative, reproducible, and large-scaled manners (19).

Here, we present a microfluidic system mimicking central-peripheral innate immunity in AD for the first time. We identified IL6, IL8, CCL2, CCL3, and CCL5 as mediators released by soluble Abeta-stimulated microglia. We validated their contribution to neutrophil recruitment by inhibiting with neutralizing antibodies. Moreover, the recruited neutrophils induce the secretion of inflammatory factors such as MIF and IL2, which may further stimulate the glia cells. Taken together, these studies suggest that central innate immune cells have a critical role in recruiting peripheral immune cells and mitigating AD pathogenesis.

## Results

### Replicated AD Microenvironment by Using a Chemotactic Microfluidic Platform

**Figure 1:**
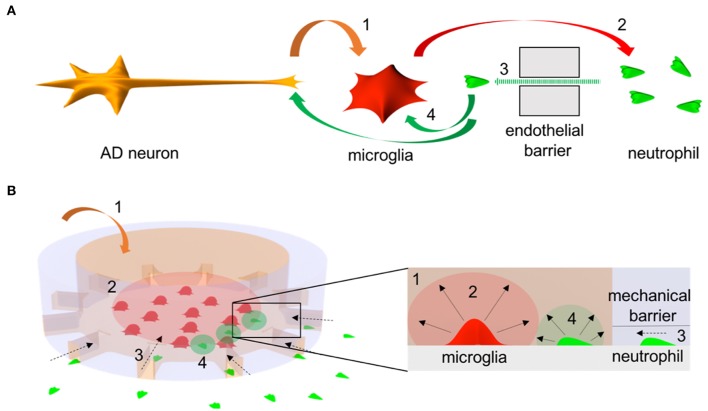
Schematics of microglia-neutrophil crosstalk in Alzheimer's disease (AD) brains and a microfluidic mimicry. **(A)** Schematics describe orchestral and multicellular crosstal k in AD brains. AD neurons secrete pathological soluble factors (1) including Abeta peptides which activate microglial proinflammation. Microglial proinflammatory soluble factors (2) disrupt a cerebral endothelial barrier and induce neutrophil chemotaxis (3). Recruited neutrophils secrete proinflammatory factors (4) that affect microglia and/or AD neurons. **(B)** Schematics describe a mimicry of the crosstalk between central-peripheral innate immune cells in our microfluidic neuroinflammatory model. To reconstruct microenvironments in AD brains, a signature molecule in AD, soluble Abeta (1) is added to a central microglial microcompartment which represents a brain parenchyma and stimulates microglia to secrete neutrophil chemoattractants (2). As a result, neutrophils in an annular microcompartment migrate across a mechanical barrier (3) representing the endothelial barrier and release additional proinflammatory factors (4) in the microglial compartment after recruitment.

We established an *in vitro* system to study the crosstalk between microglia and neutrophils in the context of AD (Figure 1). The system consists of two compartments: central circular and surrounding annular compartments representing regions of cerebral tissues and blood streams, respectively. The two compartments are connected with microchannels (10 × 5 × 500 μm^3^ in width, height, and length) representing a mechanical barrier between two regions, which only chemoattracted neutrophils can penetrate through. The long and thin migration channels serve as mechanical constraints to avoid spontaneous entrance of inactivated neutrophils and activated microglia. Firstly, we cultured human adult microglia cells in the central compartment in addition of soluble Abeta for 3 days to induce the release of microglial inflammatory mediators in the context of AD. Later, we plated freshly isolated human neutrophils on the annular compartment under effective gradients of microglial soluble factors from the central compartment. We tested 200 independent conditions on eight arrays of twenty-five devices in single well plates and measured neutrophil mobility at a single cell resolution in a real-time.

### Assessment of Microglial Neuroinflammation Activated by Soluble Abeta

**Figure 2:**
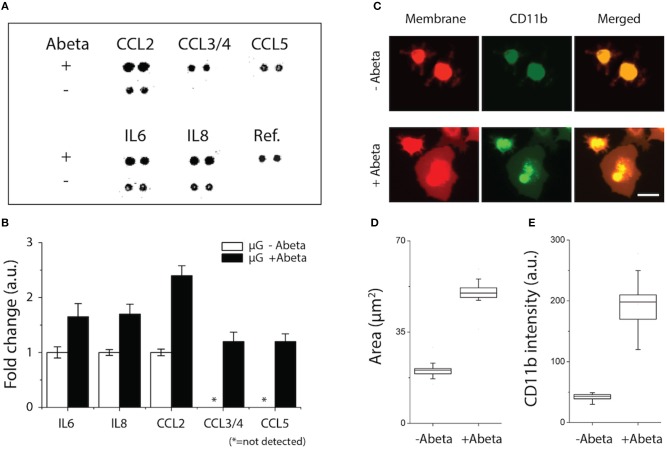
Activation of microglial proinflammation with Abeta: chemokine release, morphogenesis, and marker expression. **(A,B)** Five chemokines among 29 membrane human cytokines measured were upregulated from microglia stimulated with Abeta at 22 nM for 24 h (upper row) compared to an unstimulated condition (lower row). **(C)** Membrane-staining (red) shows the discernable change in microglial morphology with Abeta stimulation from a branch-ramified shape (resting) to an amoeboid shape (activated). A microglia activation marker of CD11b (green) was upregulated with Abeta stimulation. Both the membrane area **(D)** and the amounts of expressed CD11b **(E)** increase in the activated microglia with Abeta. n_device_ = 3, n_cell_ = 150. Scale bars: 30 μm. All parameters are presented as mean ± SEM.

We assessed the status of microglial inflammation by measuring inflammatory cytokines. We found that proinflammatory mediators, CCL2, IL-6, and IL-8 increase significantly by 1.4-, 1.9-, and 2.3-folds, respectively and chemokines, e.g., CCL3/4, CCL5 released from activated microglia only under stimulation of Abeta (Figures 2A,B). Secretion of anti-inflammatory markers, such as IL-1RA, IL-4, IL-10, and TGF-ß were below the limit of detection. These data collectively suggest that soluble Abeta induces microglial proinflammation quantified by using our microfluidic platform.

To identify the microglial activation, Abeta-treated microglial cells were monitored for three days using a time-lapse imaging microscopy. At the early 24 h, microglia remained as branched filopodia in all directions, morphological features typically associated with “resting” microglia (Figure 2C, upper, −Abeta). At 48 h after cell seeding and Abeta incubation, microglia cells became larger (Figure 2C, lower, +Abeta) and transformed into an amoeboid shape while the length and area of microglial somata increased in all directions (Figures 2C,D). In addition, microglia treated with soluble Abeta showed up-regulation of microglial activation markers such as CD11b and CD68 (Figures 2C,E, Supplementary Figure 1).

### Validation of Microfluidic Chemotaxis for Neutrophils

**Figure 3:**
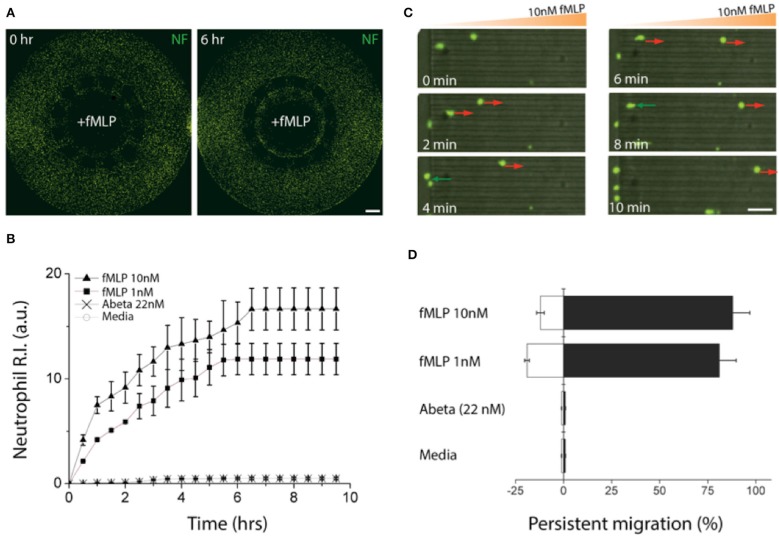
Validation of a chemotactic microfluidics for neutrophils. **(A)** Neutrophils (green) instantly migrated through microchannels size-exclusive for activated neutrophils along a gradient of fMLP (10 nM) and accumulated in a central compartment, the source of fMLP. **(B)** Time-lapse images show chemotactic migration of neutrophils by fMLP on a single cellular level. The red arrows indicate persistent chemotaxis of neutrophils along the gradient of the chemoattractant and green arrows indicate the retrotaxis against the gradient. **(C)** Counting the accumulated neutrophils in the central compartment for the first 6 h shows persistent chemotactic behavior of neutrophils responding to fMLP (10 nM, 1 nM), Abeta (22 nM), and a negative control (medium). **(D)** Measuring the migration persistence for the first 1 h shows the dominancy of the forward migration of neutrophils. Combined data from n_device_ = 3, n_neutrophil_ = 250 independent experiments. Scale bars: **(A)** 500 μm, **(B)** 50 μm. Data represented as mean ± SEM.

We induced gradients of chemoattractants for neutrophils from the central toward the annular compartments and validated the design of microchannels effective for measuring chemotactic activities of neutrophils. We defined a neutrophil recruitment index, R.I. (Figure 3A, Supplementary Figure 2), representing the fraction of neutrophils recruited to the central compartment by chemoattractants. We assessed the R.I. values under various conditions (fMLP: N-formyl-methionyl-leucyl-phenalanine, Abeta, and culturing medium) and observed discernable neutrophil recruitment only in addition of fMLP to the central compartment (Figures 3A,B). In this study, we used fMLP for a positive control as fMLP triggers p38 pathway overriding PI(3)K and is certain for effectiveness of neutrophil chemoattraction (20). However, neutrophil recruitment was not discernable with Abeta only and the medium during the observation for 6 h. The results successfully demonstrate that our platform can engage neutrophils with chemical cues actively while avoiding spontaneous entrance of neutrophils with the mechanical barriers. Neutrophil recruitment reached the peak activity at 18 ± 5 μm/min within the first 6 h (Supplementary Figure 3) and the activity was proportional to the concentration of fMLP, such as 1.6 times faster at 10 nM fMLP compared to 1 nM fMLP (Figure 3B).

We further analyzed the migratory activities of human neutrophils in a single cellular level. We found that 88.5 ± 3.8% of active neutrophils migrated forwards and 11.5 ± 1.7% migrated backward 10 nM fMLP, whereas 82.2 ± 3.4% migrated forward and 17.8 ± 2.8% backward 1 nM fMLP showing the persistent migration (Figures 3C,D). However, too much fMLP of 1 μM resulted in a 63 % reduction in the total fraction of migrating cells compared to 10 nM (Supplementary Figure 4).

### Inducement of Neutrophil Recruitment by A-Beta Activated Microglia

**Figure 4:**
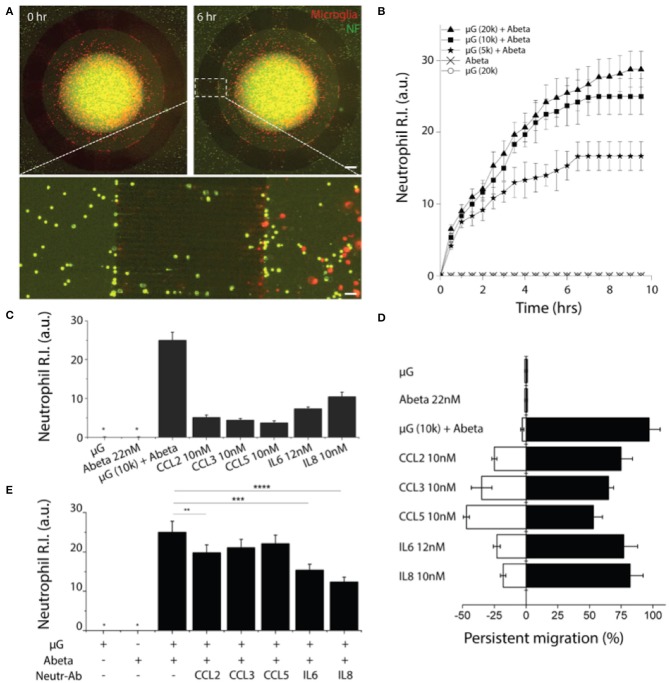
Reconstruction of human neutrophil recruitment mediated by human microglia in AD**. (A)** Fluorescent images show discernable neutrophil recruitment (green) modulated by Abeta-stimulated microglia (red). **(B)** Measurement of single cellular migration shows instant activation and saturation of neutrophil migration responding to co-culturing conditions: microglia (μG) of 20,000 cells per device, 10,000 cells per device, 5,000 cells per device with Abeta at 22 nM, and negative controls (Abeta only, 20,000 microglia only). Neutrophil chemotaxis was assessed with various soluble factors: Abeta at 22 nM, CCL2 at 10 nM, CCL3 at 10 nM, CCL5 at 10 nM, IL6 at 12 nM, and IL8 at 10 nM by counting the accumulated neutrophils **(C)** and measuring the persistence **(D)**. **(E)** The effectiveness of microglia-derived chemokines was confirmed by showing the reduced neutrophil accumulation to Abeta-stimulated microglia in addition of neutralizing antibodies for individual chemokines. Statistical significance is denoted by **P < 0.20, ***P < 0.10, ****P < 0.01 n_device_ = 4 **(B–E)**, n_device_ = 3, n_neutrophil_ = 250. Scale bars: 500 μm (upper right), 50 μm (lower pannel). All parameters are presented as mean ± SEM. *not detected.

To probe the crosstalk between microglia and neutrophils in the context of AD, we cultured microglia cells in the central compartment at various cell numbers (5,000, 10,000, 20,000 cells per device) with the treatment of soluble Abeta42 (22 nM) for 48 h, and then introduced neutrophils to the surrounding compartment. Microglia cells were pre-labeled with a red membrane dye to monitor their morphogenesis. Microglia were incubated alone for 24 h with reduced 1% FBS PrigrowIII medium without Abeta. Followed by microglial incubation with soluble Abeta, human neutrophil labeled with a green membrane dye were plated on the annular compartment. Neutrophil immediately started to migrate toward the central compartment of microglia of 5,000 with Abeta (Figure 4A). With the increase of microglia in the central compartment, neutrophil migration became more discernable and the number of recruited neutrophils increases proportional to the microglia, presuming that A-beta activated microglia release neutrophil chemoattractants. The responding neutrophils showed the enhanced polarity and distinct trailing edge toward microglial compartment with Abeta (Figure 4A, Supplementary Figure 5, Supplementary Videos 1, 2). However, neutrophil migration and the basal cell polarity were not noticeable in the conditions of microglia only, Abeta only, nor culture medium only (Figure 4B, Supplementary Video 3).

To confirm neutrophil activation, neutrophils co-cultured with microglial cells with or without Abeta for 5 min, were thoroughly washed away and the numbers of adherent neutrophils were counted. It showed that neutrophils co-cultured with Abeta-activated microglia enhanced cellular adhesion to the surface, confirming the neutrophil activation (Supplementary Figure 6).

### Identification of Microglial Chemoattractants That Induce Neutrophil Recruitment

We investigated which of microglial soluble factors are responsible for neutrophil recruitment. Firstly, we measured multiple cytokines in conditional media of Abeta-activated microglia and tested neutrophil chemotaxis for selected cytokines: IL-6, IL-8, CCL2, CCL3/4, and CCL5 (Figure 4C). We compared the recruitment activity by counting the neutrophil R.I. and found the significant recruitment by IL8 at 10 nM (R.I. = 12 ± 2.2), IL6 at 12 nM (R.I. = 8.7 ± 1.1) and discernable recruitment by CCL2 (R.I. = 5.1 ± 0.72), CCL3 (R.I. = 4.9 ± 0.51), CCL5 (R.I. = 4.2 ± 0.31) at 10 nM, respectively.

We also assessed the persistent movement of neutrophils by utilizing a single-cellular, high-resolution, and spatiotemporal imaging microscope. Neutrophils showed the highest persistent migration (~92%) toward Abeta-stimulated microglial cells, compared to single soluble factors of IL8, IL6, CCL2, CCL3, and CCL5 (79.2, 75.2, 73.1, 64.2, and 51.2%, respectively) (Figure 4D). Such the high persistency was consistent with the variation in microglial cell numbers.

### Validation of Microglial Chemoattractants That Induce Neutrophil Recruitment

To confirm the contribution of microglia-derived cytokines to neutrophil recruitment, we inhibited neutrophil migration toward Abeta-activated microglia by adding individual neutralizers for CCL2, CCL3/4, CCL5, IL6, and IL8 to the central compartment. We observed the discernable reduction of neutrophil recruitment by neutralizing antibodies against IL8, IL6, and CCL2 (Figure 4E). As reported in literature, IL8 appears to be the most dominant chemoattractant among others. IL8 neutralization reduced neutrophil recruitment most significantly by 50.2%, while IL6 (34.1%), CCL2 (22.1%), CCL3 (9.1%), and CCL5 (7.9%) neutralizers showed moderate reduction of neutrophil recruitment, respectively (Figure 4E).

### Assessment of Neutrophil Inflammation Activated by Microglial Soluble Factors

**Figure 5:**
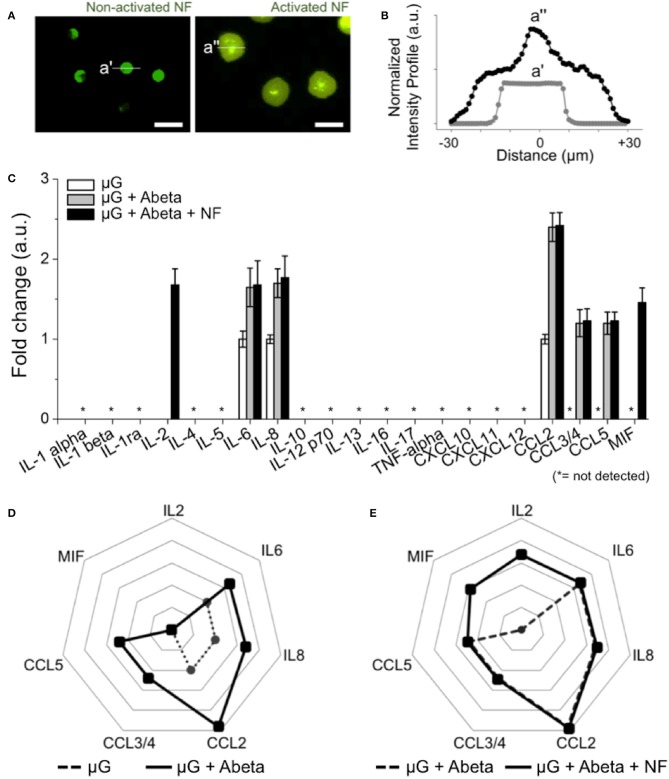
Inducement of additional proinflammatory mediators from recruited human neutrophils. **(A)** Fluorescent images show the discernable morphology change of activated neutrophils (central compartment) compared to non-activated neutrophils (annular compartment). **(B)** Intensity profile shows the significantly enlarged neutrophils after recruitment (black dotted line) compared to non-activated ones (light gray dotted line). **(C)** 21 human cytokines/chemokines for inflammation-related soluble factors were measured. Among them, MIF and IL2 are the additional soluble factors secreted from recruited neutrophils in comparison with other conditions. **(D)** A radar plot highlights the increase of IL6, IL8, CCL2, CCL3/4, CCL5 from Abeta-stimulated microglia (dotted black line) in comparison with resting microglia (dotted gray line). **(E)** A radar plot highlights the increase of MIF and IL2 from Abeta-stimulated microglia and recruited neutrophil (dotted black line) in comparison with Abeta-stimulated microglia only (dotted gray line). n_device_ = 5 **(A–E)**. Scale bars: 25 μm. All parameters are presented as mean ± SEM. *: not detected.

To assess the roles of recruited neutrophils in the progression of AD pathology, we investigated neutrophil inflammation. We compared the morphology of neutrophils resting in the annular compartment (Figure 5A left) and activated in the central compartment (Figure 5A right). Microglia-activated neutrophils extended their bodies by 1.8 folds (Figure 5B). In a 37-human cytokine assay, we found two additional cytokines, MIF and IL2 (1.7-fold and 1.67-fold, respectively) from the recruited neutrophils compared to A-beta activated microglia only (Figures 5D,E). The other 5-soluble factors from Abeta-activated microglia remained unchanged after neutrophil recruitment. Other key proinflammatory and neurotoxic cytokines including interleukin 1β (IL-1β), interleukin 17 and tumor necrosis factor alpha (TNF-alpha) were below the detection limit of the kit used (Figure 5C). This measurement suggests that the recruitment of neutrophils may exacerbate microglial neuroinflammation activated by Abeta in AD.

## Discussion

Microglial cells are cerebral innate immune cells having neuroinflammatory roles for AD pathology. Based on our previous study of neutrophil accumulation on Abeta plaques in AD animal models (17), we hypothesized that neutrophils, the frontline of peripheral immune cells are engaged with neuroinflammation not directly with Abeta but mediated by microglia. Our microscale platforms enabled comprehensive studies of the crosstalk between central and peripheral immunity and overcame significant limitations of previous studies (21–23). For example, the study of neutrophil involvement in previous models lack control over the complex cellular interactions among multiple types of brain cells and leukocytes (23). Another key feature of our platform is the capability of isolating activated neutrophils from resting neutrophils, which allows selective monitoring the activities of interest in the AD progression (21) and on-chip investigation of drug efficacy, such as, inhibitor treatment against neutrophil recruitment and microglial inflammation (23).

One of the intriguing findings is that several mediators contribute to the robust recruitment of human neutrophils, which is similar to the findings in AD mouse models (24, 25). CCL3 has been found to be associated with AD (26, 27), reported as expressed by neurons and microglia in post-mortem brains from AD patients (28) and upregulated in experimental models mimicking both amyloid and Tau deposits (29). In addition, a CCL3 polymophism has been associated with AD (30) and CCL3 secretion was found dependent on apoplipoprotein ε4, the greatest genetic risk factor for sporadic AD (31). CCL5 is most commonly involved in AD neurodegenerative processes, which regulates the activities of normal T cells. During AD pathogenesis, an elevated level of the glial CCL5 chemokine is observed in the microcirculatory system of the brain (32). Its elevated levels contribute to the recruitment of immune-competent cells, which occurs concurrently with increased rates of neuronal deaths (33). In addition to these factors, we identified two proinflammatory-specific proteins released during the crosstalk in between activated microglia and neutrophils. IL2 and MIF are known to enhance human-neutrophil inflammatory activities (25). Especially, MIF (macrophage inhibitor factor), was observed in serial brain sections of transgenic APP AD mice and stained MIF immunolabeling in neutrophils in association with Abeta plaques in the transgenic mouse brain sections (34). In addition, functional studies in murine and human neuronal cell lines revealed that Abeta-induced toxicity could be reversed significantly by a small amount of MIF, showing the beneficial effect in AD mediated by microglia (25). Finally, MIF levels in the brain cytosol and cerebrospinal fluid (CSF) of AD patients showed marked increase compare to and age-matched controls. MIF are regarded as an important proinflammatory mediator by observing in the brain cytosol and cerebrospinal fluid (CSF) of AD patients and affecting tau hyper-phosphorylation, which is found in the late stage of AD (35). IL-2 was found to attenuate Abeta pathology and synaptic impairment in AD mice by engaging astrocytes for clearing Abeta and consequently improving synaptic plasticity (36).

## Conclusion

We developed a microfluidic platform to explore the crosstalk between microglia and neutrophils in the context of AD and suggested how peripheral immune cells, neutrophils are involved in AD pathology mediated by Abeta-activated microglia to enhance the beneficial neuroinflammation. Our platform will serve as a physiologically relevant *in vitro* AD brain model for the study of neuroinflammation and a reliable tool for the validation of therapeutic strategy for neuroinflammatory immunotherapy.

## Methods

### Cell Lines, Media, and Reagents

Immortalized human adult microglia cells (SV40-microglia) were created by a company (ABM Inc., Montreal, Canada). The cells were plated onto T25 cell culture flasks (BD Biosciences, San Jose, CA, USA) and maintained in Prigrow III Medium (ABM Inc) supplemented with 10 % FBS (Life Technologies) and 1 % Pen/Strep (Invitrogen) in a CO_2_ cell culture incubator. The cell culture medium was changed every 3 days until cells were confluent.

For neutrophil isolation, human peripheral blood samples from healthy volunteers, aged 18 years and older, were collected by a company (Zen-Bio Inc., Research Triangle Park, NC, USA). Peripheral blood was drawn in a 10-mL tube containing a final concentration of 5 mM EDTA (Vacutainer; Becton Dickinson). Nucleated cells were isolated using a HetaSep gradient, followed by the EasySep Human Neutrophil Enrichment Kit (STEMCELL Technologies, Vancouver, Canada) according to the manufacturer's protocol.

### Microfluidic Device Fabrication

Negative photoresists, SU-8 5 and SU-8 100 (MicroChem, Newton, MA, USA), were sequentially patterned using standard lithography on a 4” silicon wafer to create a mold for cell migration channels of 5 μm in height and chemokine compartments of 100 μm in height. A mixture of a base and a curing agent with a 10:1 weight ratio (SYLGARD 184 A/B, Dow corning, Midland, MI, USA) was poured onto the SU-8 mold and cured for 1 h at room temperature under vacuum and, subsequently, cured for more than 3 h in an oven at 80°C. The cured poly dimethyl-siloxane (PDMS) replica was peeled off from the mold and holes were punched for fluid reservoirs. Arrayed holes were also laser-cut (Zing 24, Epilog Laser, Golden, CO, USA) into an acrylic plate of 6 mm in thickness. The machined membrane and the plate were glued together using uncured PDMS and incubated at 80°C overnight. This assembly was irreversibly bonded first to the PDMS replica using oxygen plasma at 50 mW, 5 cm, for 30 s (PX-250, March Plasma Systems, Petersburg, FL, USA), and later to a glass-bottomed single well plate (P384G-1.5-10872-C, MatTek Corp., Ashland, MA, USA). Immediately after the bonding, 10 μL of poly (l-lysine) solution (PLL, M.W. 70,000–150,000, 1.0 mg/mL, Sigma-Aldrich Co. LLC) was injected into each platform and incubated for 2 h at a room temperature to promote cellular adhesion. PLL-treated surface was rinsed with autoclaved and 0.2 μm filtered water (AM9920, Life Technologies).

### Membrane Staining of Microglia

Before the experiment, cells were washed using a serum-free medium and the cell membrane was labeled with red fluorescent dyes (PKH26PCL Red Fluorescent Cell Linker, Sigma-Aldrich). Briefly, after centrifugation (1,200 rpm.g for 5 min), the cells were re-suspended in 1 mL of Diluent C (G8278, Sigma-Aldrich) and immediately mixed with 4 μL of dye solution. The cell/dye mixture was incubated at room temperature for 4 min and periodically mixed by pipetting to achieve a bright, uniform, and reproducible labeling. After the incubation, the staining was stopped by adding an equal volume (1 mL) of 1 % BSA in PBS and incubating for 1 min to remove excess dye. Unbound dye was washed by centrifugation and suspending cells in culture medium (10^6^ cells/mL). Ten μL of the cell suspension was injected into each platform and 100 μL of a culturing medium was added into side and central extra wells. The loaded micro-devices were then incubated at 37°C supplied with 5% CO_2_.

### Isolation and Membrane Staining of Neutrophils

Human neutrophils were isolated from fresh human blood samples within 2 h of blood collection by using EasySepHuman Neutrophil Enrichment Kits (STEMCELL Technologies) and following the manufacturer's instructions. After isolation, the neutrophils were washed using 1x PBS, and immediately mixed with 4 μL of dye solution (PKH67 Green Fluorescent Cell Linker, Sigma-Aldrich). The cell/dye mixture was incubated at room temperature for 4 min and periodically mixed by pipetting to achieve a bright, uniform, and reproducible labeling. After the incubation, the staining was stopped by adding an equal volume (1 mL) of 1% BSA in PBS and incubating for 1 min to remove excess dyes. Unbound dyes were washed by centrifugation and suspending cells in culture medium (10^6^ cells/mL). Ten μL of the cell suspension was injected into each annular compartment. The loaded microfluidic-device were then incubated at 37°C supplied with 5% CO_2_ in 10 min for the cell attachment and used later in microscope.

### Time-Lapse Imaging

To track individual cells at a high throughput, we prepared 24 devices on a single-well plate and time-lapse imaged the entire devices in a large-area mode of 8 × 8 mm^2^ with a 15 % stitching by using a fully automated microscope (Ti-E/NIS Elements, Nikon Inc.) integrated with a heated incubating stage (INU-TIZB-F1, Tokai Hit Co., Ltd., Shizuoka, Japan), set at 37.5°C and 5% CO_2_ with moderate humidity. We imaged cells at every 15 min for 6 h in three channels—FITC, Cy5, and a bright field—with a 10x objective lens and a perfect focusing system in a phase contrast mode.

### Immunostaining

Before immunofluorescent staining, we rinsed the cells twice with DPBS. To fix, cells were incubated in fresh 4% paraformaldehyde aqueous solution (157-4, ElectronMicroscopy Sciences) for more than 15 min at RT followed by rinsing twice with DPBS. To permeabilize, cells were incubated in 0.1% Triton X-100 in PBST (phosphate buffered saline with 0.1% tween®20) for 15 min at RT. To block, cells were incubated in 3% human serum albumin for overnight in PBST at 4°C. After incubating with the primary antibody solutions for 24 h at 4°C, the cells were washed five times. The following antibody (and dilutions) were used: anti-cd11b (1:100, Life Technologies).

### Multiple Human Cytokine Array Kit

A human cytokine array kit was purchased from R&D Systems (Catalog # ARY005, Human Cytokine 37-membrane kit array) and utilized following the protocol provided by the manufacturer. Capture antibodies have been spotted in duplicate on nitrocellulose membranes. Cell culture supernatants are mixed with a cocktail of biotinylated detection antibodies. The sample/antibody mixture is then incubated with the array. Any cytokine/detection antibody complex present is bound by its cognate immobilized capture antibody on the membrane. Streptavidin-Horseradish Peroxidase and chemiluminescent detection reagents are added. A signal is produced, proportional with the amount of cytokine bound. Chemiluminescence is detected in the same manner as a Western blot and the resulting cytokine release profiles were quantified with ImageJ.

### Microglial Stimulation by Soluble Abeta

After stabilizing microglia in the central compartment, the culturing medium was replaced by a medium containing Abeta 42 (PAM-4349-v, Amyloid Beta-Protein, Peptides International, Inc.) at 22 nM of 2% FBS and microglia were incubated for 72 h.

### Statistical Analysis

Data, expressed as mean ± SEM, were compared using either a two-tailed Student's *t*-test when comparing two groups/conditions or one-way ANOVA followed by a *post hoc* test when comparing 3 or more groups/conditions. *P* < 0.05 was considered significant.

## Data Availability

The datasets generated for this study are available on request to the corresponding author.

## Author Contributions

JP and HC designed, fabricated, and tested devices, designed experiments, performed immunostaining, statistical quantification, generated figures, wrote and edited the manuscript. JP, SB, IM-J, DI, and HC conceived the ideas and edited the manuscript. All authors read and edited the manuscript extensively.

### Conflict of Interest Statement

The authors declare that the research was conducted in the absence of any commercial or financial relationships that could be construed as a potential conflict of interest.
