# Supplementary material for: Mimicry of Central-Peripheral Immunity in Alzheimer's Disease and Discovery of Neurodegenerative Roles in Neutrophil
Source: Front Immunol. 2019 Sep 25;10:2231. doi: 10.3389/fimmu.2019.02231 (PMC6776120; doi:10.3389/fimmu.2019.02231)
Supplement: Supplementary file 1 [file Data_Sheet_1.pdf]

Supplementary information

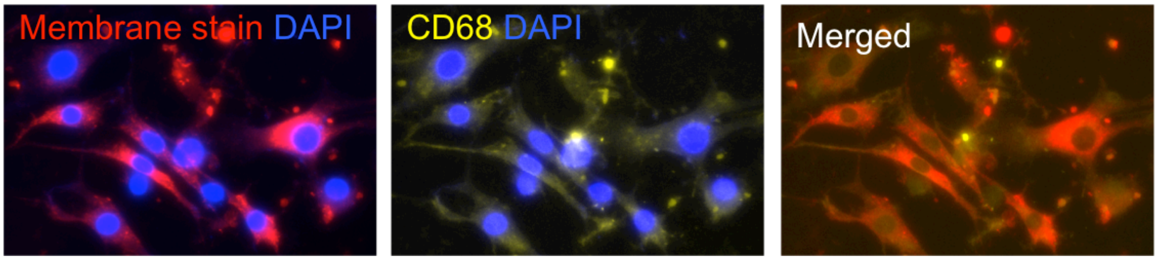

Supplementary Fig.1 CD68 immunofluorescence stain on A-beta stimulated microglial cells. Membrane stained microglial cells (red) immunostained with anti-CD68 to observe activation of microglial cells with A-beta.

R. I. = $((CC_{Day\ i} - CC_{Day\ 0}) / AC_{Day\ i})$* 100

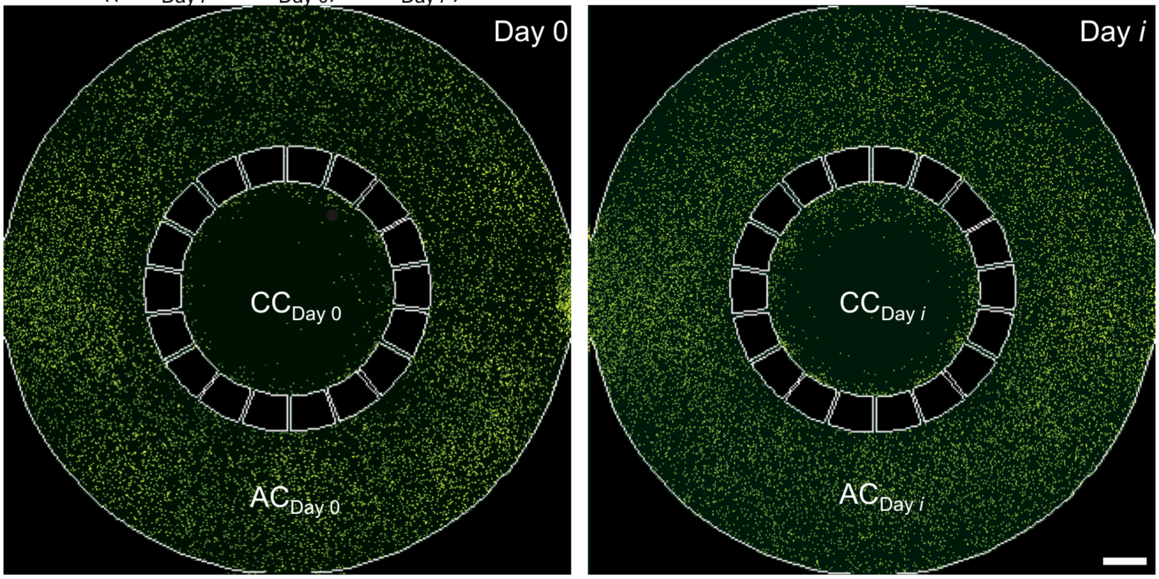

Supplementary Fig.2 Schematic representation for the definition of the recruitment index, R.I. Neutrophil recruitment was quantified by comparing the fraction of cells inside the central chamber (CC) to the total number of cells in the corresponding angular chamber device (AC). The R.I. is calculated as R.I. = $(CC_{Day\ i} - CC_{Day\ 0}) / AC_{Day\ i}$, where Day *i* is the number of cells in the central chamber and $AD_{Day\ i}$ is the number of cells in angular chamber. The recruitment index, R.I., is obtained by normalizing with the subtraction at '$CC_{Day\ 0}$' cell numbers.

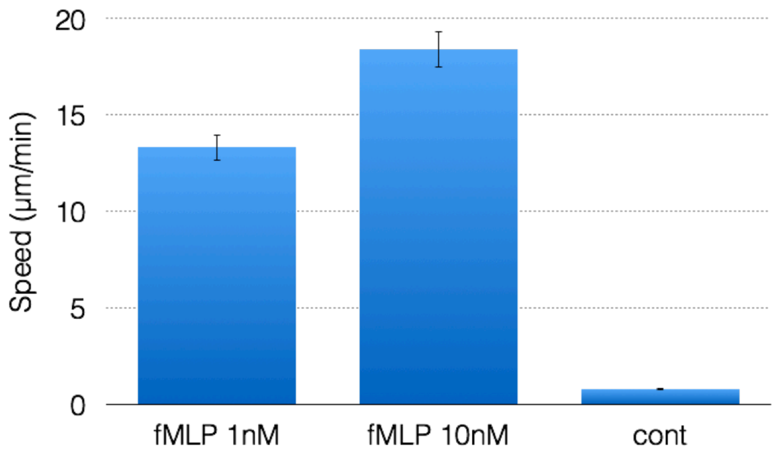

Supplementary Fig.3 Migratory speed of neutrophils migrating towards fMLP in migration channels. Neutrophils migrate faster towards in 10nM fMLP. Bars represent mean± S.D.

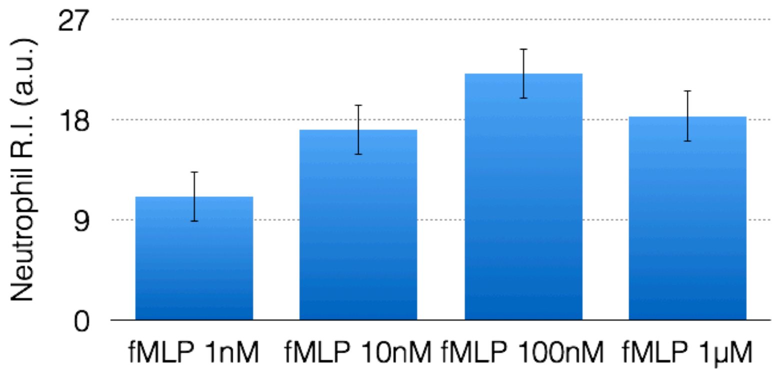

Supplementary Fig.4 Percent of human neutrophils that migrate towards fMLP at different concentrations. Increasing fMLP concentrations from 1nM to 1 μM resulted in a saturation at 100 nM and decreased at 1μM. Bars represent mean±S.D.

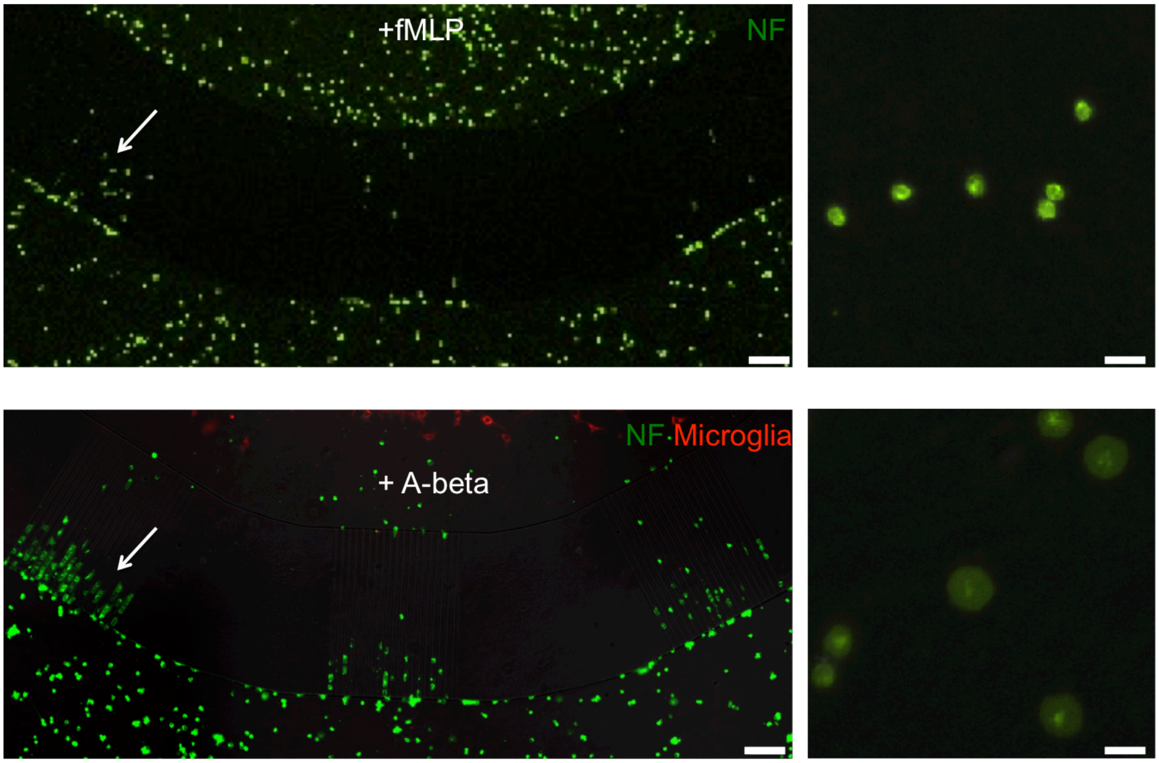

Supplementary Fig.5 Polarized neutrophil observed in co-culture condition of microglial cells stimulated with A-beta. The formation of polarized circular lamellipodia of neutrophil in co-culture condition of microglial cells (bottom) and neutrophils incubated with 100 nM fMLP (top) imaged by microscopy. Images are representative of multiple cells from four independent experiments. Scale bars, 100 µm (left column) 20 µm (right column).

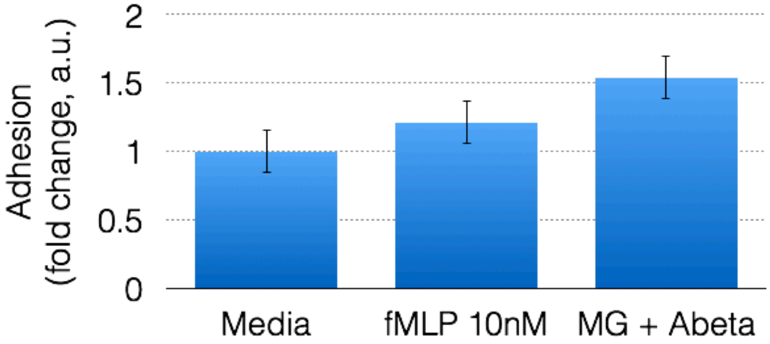

Supplementary Fig.6 Increased neutrophil attachment observed in co-culture condition of microglial cells stimulated with A-beta. Microglia mediated soluble factors increases cell adhesion to a fibronectin–coated device. Human neutrophils were uniformly distributed on angular chamber and the numbers of cells that became detached from the surface were counted. Adhesion was calculated as inversely proportional to the number of detached cells.

Video captions

Video 1. Abeta-activated microglia recruit neutrophil 1

Video 2. Abeta-activated microglia recruit neutrophil 2

Video 3. Non-activated microglia with neutrophil
